# Efficacy for the Annual Relapse Rate after the Immunosuppressive Therapy in Patients Associated with Anti-AQP4 or Anti-MOG Antibody-Positive Optic Neuritis

**DOI:** 10.1155/2020/8871146

**Published:** 2020-11-17

**Authors:** Sotaro Mori, Takuji Kurimoto, Yusuke Murai, Kaori Ueda, Mari Sakamoto, Norio Chihara, Yuko Yamada-Nakanishi, Makoto Nakamura

**Affiliations:** ^1^Division of Ophthalmology, Department of Surgery, Kobe University Graduate School of Medicine, Kobe, Japan; ^2^Division of Neurology, Department of Internal Medicine, Kobe University Graduate School of Medicine, Kobe, Japan

## Abstract

**Purpose:**

Although oral prednisolone is the first-line treatment for preventing recurrent optic neuritis (ON) after the completion of acute-phase treatment, especially anti-aquaporin 4 (AQP4) antibody-positive ON, and anti-myelin oligodendrocyte glycoprotein (MOG) antibody-positive ON, some patients experience relapses. Immunosuppressants could be effective in reducing the recurrence rate for neuromyelitis optica spectrum disorder and MOG antibody-related diseases, but there have been few studies addressing this issue focusing on the changes in ophthalmic parameters. The objective of the study was to analyze the impact of off-label uses of immunosuppressants to reduce recurrent ON.

**Design:**

Retrospective observational study, clinical case series.

**Methods:**

We reviewed the medical charts of 11 cases (22 eyes) who underwent immunosuppressive therapy in Kobe University Hospital and compared the annualized relapse rate (ARR) before and after immunosuppressive therapy. We also evaluated the dosage of prednisolone, complications of immunosuppressants, and other visual functional ophthalmologic parameters.

**Results:**

Eleven cases in total had AQP4 antibody (9 cases) and/or MOG antibody (3 cases). One case was double positive for these antibodies. Nine patients received azathioprine and two received mycophenolate mofetil as an initial immunosuppressive therapy. The median duration of immunosuppressant treatment was 2.8 years. The median ON ARR before immunosuppressive therapy was 0.33, and this decreased significantly to 0 after the therapy (*p* = 0.02). The dose of prednisolone was reduced from 17.8 ± 7.1 mg/day before to 5.8 ± 2.2 mg/day after immunosuppressive therapy (*p* < 0.01). Although two patients presented with mild elevation of liver enzymes and nausea, all patients were able to continue taking the immunosuppressants.

**Conclusions:**

Immunosuppressants can potentially decrease relapses and steroid dosage in patients with anti-AQP4 or MOG antibody-positive ON without severe adverse events and the exacerbation of visual acuities.

## 1. Introduction

A recent epidemiologic survey in Japan revealed that of 531 cases of optic neuritis (ON), 23% tested positive for either anti-aquaporin-4 (AQP4) or anti-myelin oligodendrocyte glycoprotein (MOG) antibodies [1]. The negative both AQP4 and MOG antibodies called as idiopathic ON showed good response to treatment and also seldom relapsed [2]. However, cases positive for AQP4 antibodies (AQP4-ON) or MOG antibodies (MOG-ON) frequently relapse unless maintenance therapy with immunosuppressants is begun.

Our previous study [3] has demonstrated that 70% of eyes with AQP4-ON resistant to steroid pulse therapy improved by more than three lines on a logMAR converted best-corrected visual acuity (BCVA) after plasma apheresis. However, 15% of eyes had recurrence within three months of cessation of plasma apheresis even though all patients were concomitantly treated with ≥15 mg/day of prednisolone (PSL). Other previous studies have also revealed that in cases with neuromyelitis optica spectrum disorder (NMOSD), reduction of oral PSL for maintenance therapy below 10 mg increased the rate of relapse [4]. Particularly, what dosage of PSL is necessary to suppress the relapses of attacks remains undetermined. Therefore, the substitutional drugs that have more beneficial effects on the suppression of relapse with less hazardous effects than PSL are desired.

NMOSD frequently affects middle aged or older women [1, 5]. Thus, the long-term application of oral PSL inevitably causes the development of severe systemic complications to which older women are prone, such as osteoporosis and subsequent necrosis of the femoral head and spine compression fracture, as well as other common complications including hypertension, hyperglycemia, increased risk of infection, and mental problems. These side effects not uncommonly result in the cessation of PSL as the maintenance therapy, and it is also well known that they exhibit dose dependency; the higher the dose of oral PSL, the higher the onset rate of complications [6]. Thus, substituting effective immunosuppressants for PSL in NMOSD is required to reduce the side effects of PSL and maintain long-term good visual function and quality of vision.

Previous reports demonstrated that several immunosuppressants could reduce the AQP4 antibody titer and annual recurrence rate (ARR) in cases with NMOSD [7–10]. In addition, a recent multicenter cohort study and a large number of case series demonstrated that immunosuppressants suppressed recurrence of MOG-ON and encephalitis [11, 12]. Although ARR is the most commonly applied outcome for evaluating recurrence of NMOSD, a number of studies combined cases associated with myelitis and ON, affecting the overall numbers of NMOSD. In fact, there have been few studies specifically focusing on the effects of immunosuppressants on recurrence of ON.

In the present study, we investigated the effect of off-label uses of immunosuppressants on the ARR of ON and changes in ophthalmic parameters including visual acuity and retinal structure evaluated with optical coherent tomography (OCT) in cases with NMOSD who relapsed with ON despite maintenance therapy with oral PSL.

## 2. Materials and Methods

The present study was approved by the institutional review board of Kobe University Hospital (No. 190140) and adhered to the tenets of the Declaration of Helsinki.

We retrospectively reviewed the medical records of 11 patients (22 eyes) who received any immunosuppressive therapy to suppress the relapse of ON since we began to use immunosuppressants for preventing recurrent ON. We defined “immunosuppressants” as any immunosuppressive drugs except for corticosteroids. We adopted these drugs only for patients who had a first attack of ON and took oral PSL as a first-line maintenance therapy and then received additional immunosuppressants either when relapse occurred even with continued oral PSL use or when the ON attack caused severe visual dysfunction, although the acute-phase treatment of ON was applied.

The primary endpoint of the present study was the change in ARR of ON before and after immunosuppressive therapy. For the comparison of ARRs before and after the initiation of immunosuppressants, the first ON attack was not included in the number of ON relapses before the initiation of immunosuppressants. As secondary endpoints, we evaluated the following items before and after immunosuppressive therapy: the extended ARR based on the relapse number of ON, the presence of myelitis and encephalitis, the dosage of PSL, complications of immunosuppressants, best-corrected visual acuity (BCVA) converted into the logarithm of the minimum angle of resolution (logMAR), and circumpapillary retinal nerve fiber layer (cpRNFL), and ganglion cell layer + inner plexiform layer (GCL+) thickness obtained by optical coherence tomography (OCT). LogMAR BCVA for finger counting, hand motion, light perception, and loss of light perception were defined as 1.85, 2.3, 2.8, and 2.9, respectively [3]. We defined eyes with better BCVA and worse BCVA as “dominant eyes” and “nondominant eyes,” respectively, in the same individuals.

Statistical comparisons between the variables at the initiation of immunosuppressants and at the final visit were assessed using the Wilcoxon rank sum test and paired Student's *t* test by MedCalc® software (Ver19.1.3, Ostend, Belgium). The thicknesses of cpRNFL and GCL+ were measured by a spectral domain-OCT device, 3D OCT-2000® (software version 8.00; Topcon, Inc., Tokyo, Japan) [13].

## 3. Results

The characteristics of the eleven cases included in the study are summarized in Table 1. The mean age at initiation of immunosuppressive therapy was 45.4 years old. The ratio of males to females was 0 : 11, which is almost representative of the sex difference in incidence of NMOSD. Of the eleven cases, nine (81.8%) were AQP4-ON and three (27.2%) were MOG-ON, but this included one case with both AQP4 and MOG antibodies. Her clinical characteristics were very similar to those of NMOSD such as female sex and severe visual function disability caused by ON, as previously reported.

The mean number of ON attacks was 1.7 and that of myelitis and encephalitis was 0.7 at the initiation of immunosuppressive therapy. The median interval between the first onset of ON and initiation of the immunosuppressants was 1.8 years. All cases received steroid pulse therapy. Due to the insufficient effect of steroid pulse therapy, five cases received additional plasma exchange and three cases had intravenous immunoglobulin therapy for first attack or relapses. All cases received oral medication of PSL as their first-line maintenance therapy.

Table 2 shows types of immunosuppressants and their side effects. Nine patients received azathioprine and two received mycophenolate mofetil as an initial immunosuppressive therapy. There were minor complications in two cases with azathioprine. One case, who suffered from nausea with azathioprine, was switched to tacrolimus. However, digestive symptoms emerged after switching, reducing the dose by half relieved the symptoms. However, this case had an ON relapse later, recovered following plasma exchange, and did not relapse again with regular dosage of oral tacrolimus. Thus, all cases were eventually able to continue taking the immunosuppressants orally until the last visit.

**Figure 1:**
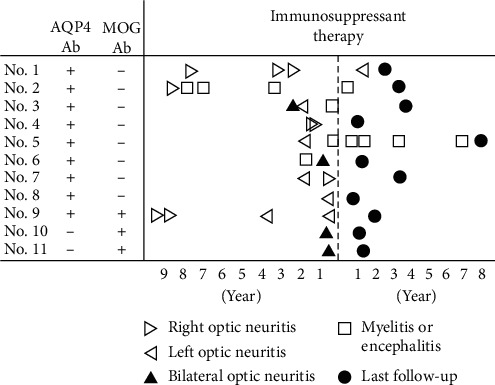
A summary of the treatment history, relapses, and treatment of all 11 patients in the study. Initial onset of ON was not included in the determination of number of relapses in the study. AQP4: anti-aquaporin4 antibody; MOG: anti-myelin oligodendrocyte glycoprotein.

Figure 1 depicts relapse profiles of ON, myelitis, and encephalitis in all cases before and after the initiation of immunosuppressive therapy. The numbers of relapses in most cases clearly decreased after the initiation of immunosuppressive therapy. During the observation period, only Case 1 relapsed with ON, and only once. The case with an ON relapse after immunosuppressive therapy was recovered by plasma exchange after the failed additional steroid pulse therapy, followed by oral half dose tacrolimus. Case 2 and Case 5 experienced myelitis once and four times, respectively.

Table 3 shows the change of ARR and ophthalmologic parameters before and after immunosuppressive therapy. The median ON ARR significantly decreased from 0.33 to 0 after immunosuppressive therapy (Wilcoxon rank sum test, *p* = 0.03). In addition, the dosage of PSL was significantly reduced from 17.8 ± 7.1 mg/day at the initiation of immunosuppressive therapy to 5.8 ± 2.2 mg/day at the final visit (paired Student's *t*-test, *p* < 0.01). The median extended ARR also significantly decreased from 0.37 to 0 (paired Student's *t* test, *p* = 0.03). The mean logMAR BCVA was not significantly different between the initiation of immunosuppressive therapy and the final visit (paired Student's *t* test, *p* = 0.26 (both eyes), *p* = 0.81 (dominant eyes), and *p* = 0.22 (nondominant eyes)). The thickness of cpRNFL and GCL + also did not change between the initiation of immunosuppressive therapy and the final visit. During the median 2.8 years of the observation period since the initial immunosuppressive therapy, there was a subtle reduction in these OCT parameters, which may simply reflect the age-related retinal atrophy or subclinical reduction associated with the disease [14, 15].

## 4. Discussion

In the present study, we found that off-label use of azathioprine and mycophenolate mofetil was effective for the suppression of ON relapse. Additionally, these drugs were well tolerated, although there were a few minor adverse events.

In the present study, the ARR before initiation of immunosuppressants was 0.33, which was lower than that of NMO without any immunosuppressive therapy (median ARR, 0.7–1.48) [4, 16]. All patients, except for cases 10 and 11 who were seropositive for anti-MOG antibody, received low-dose prednisolone monotherapy before the initiation of immunosuppressants. Although the present study included both patients with anti-AQP4 and anti-MOG antibodies, the ARR immunosuppressant initiation was presumed to be equivalent to that with prednisolone monotherapy. A study previously reported that low-dose prednisolone monotherapy decreased ARR from 1.48 to 0.49 compared to patients with NMO who did not receive prednisolone [4]. Thus, the ARR before immunosuppressant initiation is suggested to be close to that of the previous report. The low ARR before combining prednisolone with immunosuppressive therapy may reflect the effect of prednisolone monotherapy.

The question of which immunosuppressants have the most suppressive effect on relapse in NMOSD remains undetermined [9, 16–23]. Although there were differences in the background of cases in previous studies, most immunosuppressants still significantly decreased the ARR from 1–3 to 0–1 [9, 16–23]. Li et al. demonstrated that the administration of azathioprine decreased the ARR from 1.41 to 0.36 in 32 cases with NMOSD [19]. Huang et al. demonstrated that mycophenolic acid mofetil for a median duration of 18 months reduced ARR from 1.02 to 0 in 19 cases with NMSOD [23]. Several studies comparing two different immunosuppressants showed that rituximab was more effective than azathioprine [9, 10, 24–26], but a few reports showed no significant difference [27]. Other reports showed that there were no significant differences in ARR between azathioprine and mycophenolic acid mofetil [24, 28, 29]. Furthermore, Yang et al. demonstrated no significant difference between azathioprine, mycophenolic acid mofetil, and rituximab in their effectiveness at reducing ARR in a prospective cohort study [24]. By contrast, a recent network meta-analysis has shown that rituximab significantly decreased ARR compared to azathioprine and suggested that, out of rituximab, azathioprine, mycophenolic acid mofetil, cyclophosphamide, and cyclosporine A, rituximab and mycophenolic acid mofetil are recommended from a safety viewpoint [30]. In the present study, we chose azathioprine as the first-line drug because azathioprine is the cheapest in Japan and rituximab requires intravenous administration, making its long-term use practically difficult. Overall, azathioprine may be one of the most optimal immunosuppressants to apply to NMOSD cases.

However, recent randomized clinical trials have presented strong evidence that three types of humanized monoclonal antibodies exert promising outcomes to prevent relapse of NMOSD with AQP4 antibody: eculizumab, satralizumab, and inebilizumab [31–33]. Eculizumab interferes with cleavage of the complement protein C5 into C5a and C5b. C5a is a proinflammatory protein and acts as anaphylatoxin, and C5b is involved in the formation of membrane associated complex, both of which are known to be responsible for the neurodegeneration in the NMOSD pathogenesis [31]. Satralizumab inhibits the interleukin-6 receptor and prevents maturation of naïve T cells into helper T cells, which eventually reduces the maturation of B cells into plasma cells that produce the AQP4 antibody [32]. Inebilizumab binds to CD19 antigen present on the surface of B cells and exclusively depletes B cell lines [33]. Although the effectiveness of these monoclonal antibodies in NMOSD mainly target myelitis, they may be also promising to retard ON relapses in patients with the AQP4 antibody. The present study elucidated that oral immunosuppressants could decrease the recurrent ON greatly; however, two patients (patient's Nos. 2 and 5) who had been originally associated with AQP4-ON, relapsed myelitis, were resistant to azathioprine or mycophenolate mofetil. Therefore, these patients with frequent relapses of myelitis might be a good adaptation of the molecular target drugs for preventing relapse. In the future, a comparison study or an effectiveness study would be necessary to examine the efficacy of the newest molecular target drugs against patients resistant to existing immunosuppresants.

The application of immunosuppressants for MOG antibody-positive patients is still controversial. In fact, in the nationwide epidemiologic ON study in Japan, the median visual acuity after treatment of MOG-ON was almost similar to that in the MOG and AQP4 antibody negative cases including idiopathic optic neuropathy [1] (median logMAR 0 and 0.097, respectively). In contrast, Liu et al. demonstrated that 15% of cases of encephalomyelitis positive for the MOG antibody suffered from severe disability even after treatment and 4 of 22 MOG-ON cases achieved less than 1/10 visual acuity [34]. Matsuda also demonstrated that 2 of 18 cases positive for the MOG antibody were resistant to steroid pulse therapy [35]. These previous studies indicate the possibility that cases with MOG-ON include several distinct subtypes, although further studies are needed to clarify this point.

There are some limitations in the present study. First, the study is a single center and retrospective observational study. Second, the low number of cases and the short follow-up period of some cases might have affected the statistical analyses for ARR and OCT. Third, we analyzed both anti-AQP4-ON and MOG-ON together. We believe that restricting the population to one individual with a single disease is better. However, we assured that our analysis indicated the effectiveness for MOG-ON as well as AQP4-ON.

A number of clinical trials have demonstrated that the variety of immunosuppressants reduced the relapse rate for patients with NMOSD and MOG antibody-related diseases. Unfortunately, the results from most studies were based on comprehensive evaluation criteria such as expanded disability status scale. In other words, clinical studies focusing on the changes in ophthalmic parameters of these patients from the viewpoint of neuro-ophthalmologists are few. Despite the abovementioned limitations, our study findings may emphasize that the existing and classical immunosuppressants such as azathioprine and mycophenolate mofetil sufficiently suppress the relapse of attacks and simultaneously maintain visual function and decrease steroid dosage.

## Figures and Tables

**Table 1:** Summary of patient characteristics.

*At baseline*	
Number of patients/eyes	11/22
Sex (male: female)	0 : 11
Age (yrs), mean (SD)	45.4 (19.1)
Location of lesion	Only ON 8 cases, ON + myelitis and encephalitis 3 cases
Antibodies	AQP4 antibody: 9 cases
	MOG antibody: 3 cases
	Double positive: 1 case

*At the initiation of immunosuppressive therapy*	
Mean age, years (SD)	42.5 (19.7)
Mean number of ON attack episodes (SD)	1.7 (1.0)
Mean number of myelitis and encephalitis episodes (SD)	0.7 (1.4)
Median follow-up period before start of immunosuppressive therapy, years (first-third quartile)	1.8 (0.8–5.2)

*Types of treatments (number of patients)*	
Only methylprednisolone	4
Methylprednisolone + plasma exchange	4
Methylprednisolone + intravenous immunoglobulin	2
Methylprednisolone + intravenous immunoglobulin + plasma exchange	1

SD: standard deviation, ON: optic neuritis, AQP4: anti-aquaporin4 antibody, MOG: anti-myelin oligodendrocyte glycoprotein MP: methylprednisolone, PE: plasma exchange, IVIg: intravenous immunoglobulin.

**Table 2:** Immunosuppressive therapy.

Initial immunosuppressant drugs	Azathioprine 9 cases mycophenolate mofetil 2 cases
Complications	Elevation of liver enzymes 1 case nausea 1 case
Median observation period after the beginning of immunosuppressive therapy, years (first-third quartile)	2.8 (1.6–4.0)
Relapse	ON 1 case, 1 time myelitis 2 cases, 5 times

ON: optic neuritis, SD: standard deviation.

**Table 3:** The efficacy of immunosuppressive therapy.

	At the initiation of immunosuppressive therapy	At final visit
Median ARR of ON (first-third quartile)	0.33 (0–0.45)	0 (0–0)
Median ARR of ON, myelitis, and encephalitis episode (first-third quartile)	0.37 (0.17–0.55)	0 (0–0.13)
Mean PSL dosage (mg/day) (SD)	17.8 (7.1)	6.2 (2.4)
Mean logMAR BCVA (SD)		
Dominant eye	−0.1 (0.2)	−0.1 (0.2)
Nondominant eye	0.9 (1.0)	0.9 (1.0)
Mean GCL + thickness (*μ*m) (SD)		
Dominant eye	59.5 (12.5)	57.5 (10.5)
Nondominant eye	49.8 (15.3)	45.4 (14.5)
Mean cpRNFL thickness (*μ*m) (SD)		
Dominant eye	71.2 (21.0)	74.4 (18.1)
Nondominant eye	59.9 (22.1)	58.0 (13.4)

ARR: annualized relapse rate, ON: optic neuritis, PSL: prednisolone, SD: standard deviation, BCVA: best-corrected visual acuity, GCL: ganglion cell layer, cpRNFL: circumpapillary retinal nerve fiber layer.

## Data Availability

The datasets used and/or analyzed during the current study are available from the corresponding author on reasonable request.
